# Epidemiological, mechanistic, and practical bases for assessment of cardiorespiratory fitness and muscle status in adults in healthcare settings

**DOI:** 10.1007/s00421-022-05114-y

**Published:** 2023-01-23

**Authors:** Jaime A. Gallo-Villegas, Juan C. Calderón

**Affiliations:** 1 GRINMADE Research Group, Faculty of Medicine, University of Antioquia, Carrera 51D No 62-29, Medellin, Colombia; 2 Centro Clínico y de Investigación SICOR (Soluciones Integrales en Riesgo Cardiovascular), Medellín, Colombia; 3 Physiology and Biochemistry Research Group-PHYSIS, Faculty of Medicine, University of Antioquia, Medellín, Colombia

**Keywords:** Physical fitness, Cardiorespiratory fitness, Oxygen consumption, Skeletal muscle, Muscle strength, Mortality

## Abstract

Given their importance in predicting clinical outcomes, cardiorespiratory fitness (CRF) and muscle status can be considered new vital signs. However, they are not routinely evaluated in healthcare settings. Here, we present a comprehensive review of the epidemiological, mechanistic, and practical bases of the evaluation of CRF and muscle status in adults in primary healthcare settings. We highlight the importance of CRF and muscle status as predictors of morbidity and mortality, focusing on their association with cardiovascular and metabolic outcomes. Notably, adults in the best quartile of CRF and muscle status have as low as one-fourth the risk of developing some of the most common chronic metabolic and cardiovascular diseases than those in the poorest quartile. The physiological mechanisms that underlie these epidemiological associations are addressed. These mechanisms include the fact that both CRF and muscle status reflect an integrative response to the body function. Indeed, muscle plays an active role in the development of many diseases by regulating the body’s metabolic rate and releasing myokines, which modulate metabolic and cardiovascular functions. We also go over the most relevant techniques for assessing peak oxygen uptake as a surrogate of CRF and muscle strength, mass, and quality as surrogates of muscle status in adults. Finally, a clinical case of a middle-aged adult is discussed to integrate and summarize the practical aspects of the information presented throughout. Their clinical importance, the ease with which we can assess CRF and muscle status using affordable techniques, and the availability of reference values, justify their routine evaluation in adults across primary healthcare settings.

## Introduction

Poor cardiorespiratory fitness (CRF) and muscle status are associated with an increased risk of chronic disease and mortality from the fourth decade of life onward in both sexes (Kodama et al. 2009; Silventoinen et al. 2009; Leong et al. 2015; Schmid and Leitzmann 2015; Spahillari et al. 2016; de Santana et al. 2021; Qiu et al. 2021; Han et al. 2022). The CRF reflects the integrative capacity of the organism to transport oxygen from the atmosphere to the mitochondria during physical activity. For this reason, it is considered a robust indicator of the health of an individual (Kaminsky et al. 2013; Ross et al. 2016). Similarly, muscle strength, mass, and quality and performance in functional tests (Cooper et al. 2010; Cooper et al. 2014; Cruz-Jentoft et al. 2019) reflect the integrative capacity of the organism to perform physical activities where the skeletal muscle fulfills an essential function. A poor muscle status, characterized by low muscle strength, mass, and quality, and performance in functional tests, is associated with a greater risk of many chronic diseases, and mortality from all causes (Silventoinen et al. 2009; Cooper et al. 2010; Cooper et al. 2014; Lopez-Jaramillo et al. 2014; Leong et al. 2015; Cruz-Jentoft et al. 2019; Srikanthan and Karlamangla 2011; Londono et al. 2012; Chin et al. 2013; Park and Yoon 2013; Argiles et al. 2016), long before the formal criteria for the diagnosis of the disease known as sarcopenia are met. For this reason, muscle assessment should be done from early adulthood (i.e., since age 30). Skeletal muscle tissue is the main component of lean tissue reported in many studies, especially if the analyses are carried out in the extremities (i.e., appendicular mass), so in this text, and for simplicity, we will consider *lean* and *muscular* as synonyms (Wigodski et al. 2019).

Given the enormous potential of CRF and muscle status measurements as robust indicators of health status and predictors of important clinical outcomes whose evaluation and follow-up are beneficial for patients, they have become new vital signs (Bohannon 2008; Ahima and Park 2015; Ross et al. 2016). Even so, CRF and muscle status are not routinely assessed in adults in health practice (Bohannon 2008; Kaminsky et al. 2013; Ross et al. 2016; Ibrahim et al. 2018). This is likely because knowledge in the area has not reached health training programs. In the academic curricula, as well as in health practice and public health measures, there is an emphasis on the control of obesity and pharmacological interventions as strategies for health promotion and treatment of different chronic diseases (Pedersen and Saltin 2006; Kujala 2009; Gallo et al. 2010; Fletcher et al. 2018). In addition, until a few years ago, there were no reference values for multiple populations around the world against which to compare the results of simple tests of CRF and muscle status in adults. Finally, the emphasis on diagnosing and taking care of the older, certainly of paramount importance, have obscured the fact that the condition of the older is reached by walking a path which starts in the early adulthood and can be acknowledged since then (Sui et al. 2007; Cruz-Jentoft et al. 2019).

This review aims to show that the above limitations and paradigms can be overcome and challenged by recent knowledge, which now justifies the routine evaluation of the CRF and muscle status in primary healthcare settings starting the fourth decade of life, as a public health measure. The following aspects are addressed: (i) epidemiological evidence of the association of CRF and muscle status with morbidity and mortality, focused on cardiovascular and metabolic (i.e., cardiometabolic) outcomes; (ii) physiological mechanisms by which a better CRF and muscle status protect against adverse outcomes; (iii) methods to quantify CRF and muscle status; and (iv) application of concepts and translational recommendations based on a clinical case.

## Association between CRF and morbidity and mortality

### Inverse association between CRF and morbidity

In the last three decades, there has accumulated overwhelming evidence on the association between CRF and the risk of mortality and other adverse health outcomes (Blair et al. 1989; Myers et al. 2002; Kodama et al. 2009; Qiu et al. 2021; Han et al. 2022). A poor CRF is associated with an increased risk of cardiovascular disease and mortality from all causes after adjusting for age and other potential confounding variables (Kodama et al. 2009; Schmid and Leitzmann 2015; Qiu et al. 2021; Han et al. 2022). These findings have been reported in adults, asymptomatic men and women, people of different ethnic origins, and those with obesity, hypertension, type 2 diabetes mellitus, cardiovascular disease, and cancer (Blair et al. 1989; Myers et al. 2002; Church et al. 2004; Kodama et al. 2009; Kokkinos et al. 2013; Faselis et al. 2014; Ezzatvar et al. 2021a, 2021b). Additionally, a poor CRF has been associated with a greater risk of other adverse health outcomes, such as (i) cardiovascular and noncardiovascular outcomes after surgical procedures (Smith et al. 2009); (ii) time to heart transplant and hospitalization in patients with heart failure (Arena et al. 2014); (iii) incidence of stroke in older adults (Jefferis et al. 2014); (iv) dementia and Alzheimer's disease (Lee 2021); (v) metabolic syndrome and type 2 diabetes mellitus (Lee et al. 2009); and (vi) disability (Rabiee et al. 2015).

### Inverse association between CRF and mortality

Different meta-analyses reported an inverse dose–response association between CRF expressed in terms of metabolic equivalents (MET, 3.5 mL O_2_/kg/min) and the risk of death (Kodama et al. 2009; Qiu et al. 2021; Han et al. 2022). An increase of 1 MET decreases mortality from all causes (12%), cardiovascular disease (13%), and cancer (7%) (Han et al. 2022). These findings have been described in apparently healthy people (Kodama et al. 2009; Han et al. 2022), in those with established cardiovascular disease (Ezzatvar et al. 2021a), and in cancer patients (Ezzatvar et al. 2021b). Likewise, a CRF > 10 METs in adults is associated with greater survival, while a CRF < 5 METs is associated with higher mortality (Kodama et al. 2009; Han et al. 2022). The protective effect of a better CRF on mortality is independent of age, ethnicity, adiposity, smoking, alcohol consumption, and the presence of comorbidities (Kodama et al. 2009; Han et al. 2022; Harber et al. 2017; Kaminsky et al. 2019a).

### Usefulness of CRF in the reclassification of cardiovascular risk

CRF has a higher prognostic value than established classical risk factors (Myers et al. 2002; Church et al. 2004; Kodama et al. 2009; Kokkinos et al. 2013; Faselis et al. 2014) and some variables that are measured during the electrocardiographic stress test (ST-segment depression, symptoms, and hemodynamic response) (Kligfield and Lauer 2006) for the development of cardiovascular disease and mortality. Including the CRF as one of the risk factors improves the performance of prediction models of morbidity or mortality due to cardiovascular disease (Mora et al. 2005; Israel et al. 2016; Kondamudi et al. 2021). Interestingly, given that CRF is a variable that responds to treatments, its repeated measurement over time could contribute to risk stratification (Blair et al. 1995). People who increase their CRF between two measurements have a lower risk of adverse health outcomes than those whose physical fitness is steady or decreased over time (Blair et al. 1995).

Efforts have been made to incorporate CRF into predictive models of cardiovascular risk assessment (Framingham and European risk score) (Mora et al. 2005; Israel et al. 2016; Kondamudi et al. 2021) due to its potential to reclassify the risk of adverse outcomes (improvement in the net reclassification rate from one risk category to another of 10–40%) (Ross et al. 2016) and to monitor changes over time. Unfortunately, international public health organizations have lagged to include it in prevention strategies, delaying its widespread teaching and use.

## Association between muscle status and morbidity and mortality

### Inverse association between muscle status and morbidity

Studies conducted in adults have shown a negative association of handgrip strength and global or appendicular muscle mass with insulin resistance (IR), glycemic control, and the risk of developing metabolic syndrome and diabetes (Atlantis et al. 2009; Srikanthan and Karlamangla 2011; Londono et al. 2012; Park and Yoon 2013; Argiles et al. 2016; Kim and So 2016; Haines et al. 2022). For example, grip strength or global lean mass in the lowest quartile of the population is associated with up to 4 times a higher risk of developing metabolic syndrome (Atlantis et al. 2009; Yi et al. 2018). The spectrum of complications associated with lower grip strength or lower muscle mass includes an increased risk of falls, fractures, disability, infections, respiratory disease, and hospitalizations (Bohannon 2008; Leong et al. 2015; Argiles et al. 2016).

Qualitatively similar relationships have been reported between grip strength and knee extension strength and cardiovascular outcomes: the lower the strength, the greater the risk of myocardial infarction, cerebrovascular events, and heart failure (Silventoinen et al. 2009; Lopez-Jaramillo et al. 2014; Leong et al. 2015). Lower grip strength was a risk factor for falling in diabetic adults (Wen et al. 2021). The negative association of grip strength and muscle mass with blood pressure and cardiometabolic risk factors, such as pulse wave velocity and glycemic or lipidic alterations, has been observed since early adulthood (Cohen et al. 2014; Furushima et al. 2017). People with greater grip strength have better values of parameters of cardiometabolic importance, such as body mass index, waist circumference, blood pressure, and plasma glucose or lipids (Yi et al. 2018).

Low muscle quality, reported in terms of high myosteatosis, is also associated with worse cardiometabolic outcomes (Kim and Kim 2021). All the above associations are maintained even after adjusting for age, sex, adiposity or body size variables, and physical activity.

### Inverse association between muscle status and mortality

In longitudinal studies, greater grip strength or total or appendicular muscle mass has been associated with a significant reduction in mortality from cardiovascular causes and from all causes in adults and elderly adults, both healthy and with glycemic alterations, in both men and women (Bohannon 2008; Cooper et al. 2010; Cooper et al. 2014; Lopez-Jaramillo et al. 2014; Leong et al. 2015; Spahillari et al. 2016; Steiber 2016; de Santana et al. 2021). Subjects in the tertile of higher muscle density, that is, with the lowest fat infiltration and therefore a higher muscle quality, showed up to a 70% lower risk of overall mortality than the worst tertile (Larsen et al. 2020).

In relation to more integrative evaluations of muscle performance, such as sitting repeatedly in a chair, or walking speed, poorer physical capacity is associated with higher mortality from all causes in both sexes (Cooper et al. 2010; Cooper et al. 2014). For example, people in the quartile with the lowest walking speed have 3 times the risk of death as the quartile with the highest speed (Cooper et al. 2010).

The findings cited in this section apply to people between the fourth and ninth decades of life, without the diagnosis of sarcopenia, and health conditions taken from the general population. In addition, the results are maintained after multiple adjustments and analyses in countries with ethnic, cultural, and socioeconomic diversity (Leong et al. 2015), confirming that muscle status is an independent risk factor for cardiometabolic and global morbidity and mortality.

### The special case of sarcopenia

The population that is below 2.5 standard deviations of muscle strength (compared to the values in young people), accompanied by a reduction of muscle mass, meet the criteria of the most recent guidelines for the diagnosis of sarcopenia (Cruz-Jentoft et al. 2019). The reader is referred to a specialized bibliography given that the topic of sarcopenia is outside the scope of this review (Rubbieri et al. 2014; Cruz-Jentoft et al. 2019; de Santana et al. 2021).

## Biological mechanisms of CRF and muscle status as protective against adverse outcomes

### Protective cardiorespiratory mechanisms

The CRF expressed in terms of the maximal oxygen uptake (VO_2max_) reflects the integrative capacity of the organism to transport oxygen from the atmosphere to the mitochondria during physical activities (Balady et al. 2010). VO_2_ can be expressed in mL/kg/min (relative to body mass), L/min (absolute values), or in METs (Balady et al. 2010). The variability of VO_2max_ depends on age, sex, physical activity, and genetic factors (Bouchard et al. 1999; Balady et al. 2010). VO_2max_ decreases with age and is lower in women than in men and may improve after an exercise training program (Bouchard et al. 1999; Balady et al. 2010; Batacan et al. 2017; Su et al. 2019).

VO_2max_ is considered the best measure of CRF and physical work capacity (Balady et al. 2010). From Fick’s principle (VO_2_ = Q × avO_2_), where VO_2_ is oxygen consumption, Q is cardiac output, and avO_2_ is the arteriovenous oxygen difference, it can be deduced that VO_2max_ depends on the processes related to (i) ventilation and pulmonary diffusion; (ii) the functioning of the ventricles; (iii) ventriculoarterial coupling; (iv) the ability of blood vessels to efficiently transport blood from the heart to different organs according to oxygen requirements; (v) oxidative adaptations of skeletal muscle to extract oxygen from arterial circulation; and (vi) the integration of metabolic signals with the central commands of the cardiovascular system (Balady et al. 2010). In this sense, any alteration in one of the processes described could lead to a decrease in the CRF of an individual. Given the ability to integrate and demonstrate the condition of various systems, VO_2max_ as a reflection of CRF is considered a robust indicator of human health (Kaminsky et al. 2013; Ross et al. 2016).

**Fig. 1:**
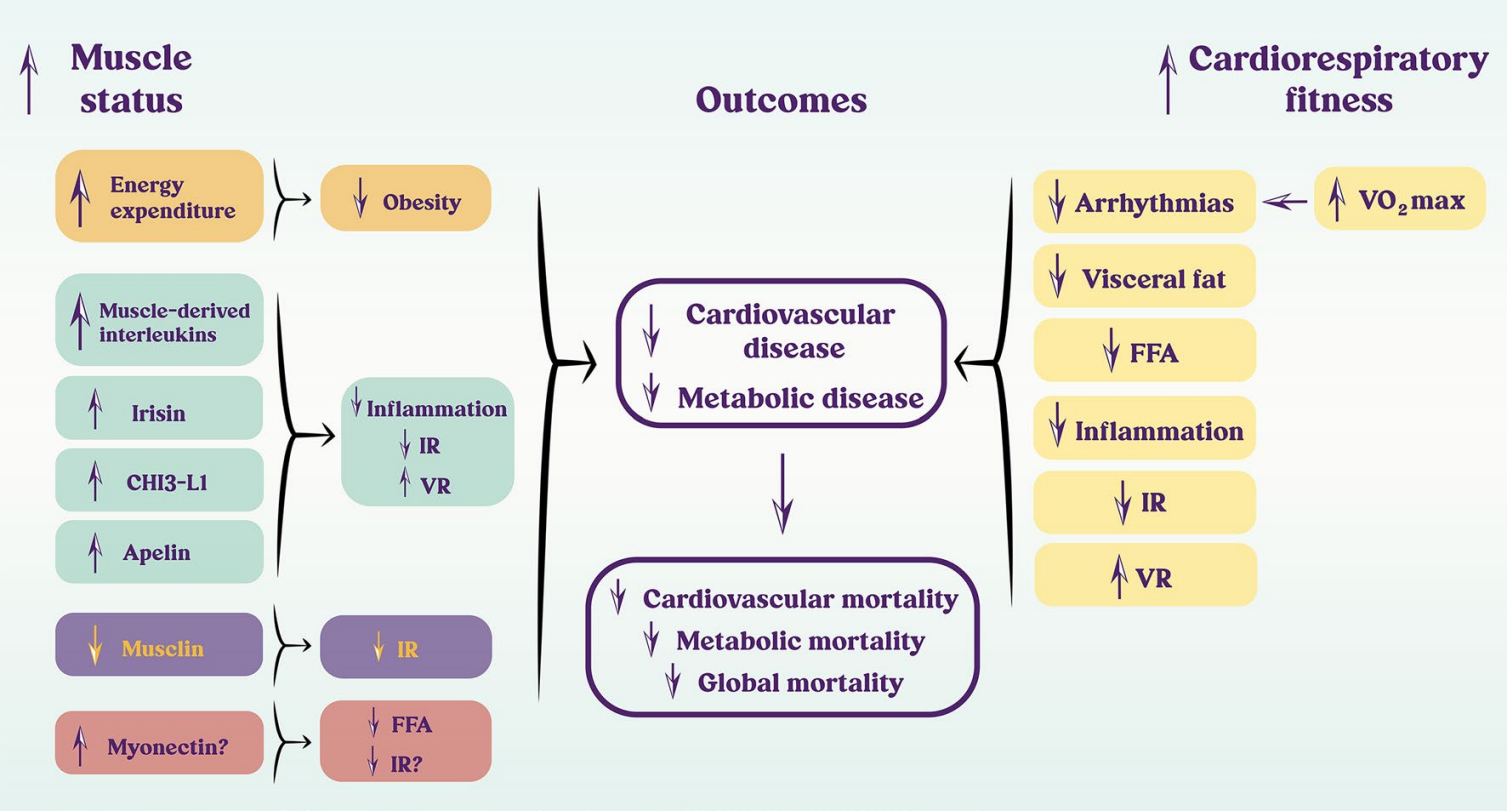
Physiological mechanisms by which better cardiorespiratory fitness and muscle status protect against adverse outcomes. A better cardiorespiratory fitness (right), as reflected by a higher VO_2_max, associates with less arrhythmias, visceral fat, free fatty acids (FFA), inflammation and thus insulin resistance (IR). This profile also favors improved vascular reactivity (VR). A better muscle status (left), as indicated by a higher handgrip and muscle mass, increases energy expenditure, and endows with a healthy profile of myokines such as muscle-derived interleukins, irisin, chitinase-3-like protein 1 (CHI3-L1), apelin and myonectin. The result is a reduction in obesity risk, FFA, inflammation and IR and the associated improvement in VR. The reduction in musclin reinforces the reduction in IR. Together, good cardiorespiratory fitness and muscle status reduce the risk of developing cardiovascular and metabolic diseases, which directly leads to a reduction in mortality

A few biological mechanisms can explain why a better CRF is a protective factor: (i) a cardioprotective cardiovascular risk profile, mediated in part by an associated greater level of physical activity; (ii) better body composition (less visceral fat and more muscle mass); (iii) more favorable lipid profile; (iv) lower blood pressure values; (v) increased sensitivity to insulin; (vi) less inflammation; (vii) a higher vagal tone, which lowers the risk of arrhythmias; (viii) lower risk of thrombotic events; and (ix) better endothelial function (Ravussin et al. 1988; Zurlo et al. 1990; Gallo et al. 2010; Wisloff et al. 2005; Fiuza-Luces et al. 2013, 2018; Fletcher et al. 2018; Pantiya et al. 2022) (Fig. 1).

### Protective muscle mechanisms

Until recently, from the metabolic point of view, skeletal muscle was considered a passive energy store/consumer and the target of molecules produced by other tissues. However, during the last two decades, it was definitively recognized that the muscle itself is a primary determinant of the health-disease relationship and that it plays an active role in the pathophysiology of cardiometabolic diseases independent of other risk factors.

Given that the relationship between muscle and morbimortality is independent of the level of physical activity (see the previous section) and that muscle strength depends on the mass and fibre type composition, the three aspects that mediate the protective role of muscle are: (i) muscle mass, (ii) its composition of fibre types, and (iii) its function as an endocrine organ. These three variables can, in an articulated way, modulate metabolic and hemodynamic aspects relevant to multiple chronic cardiovascular and metabolic diseases.

### Mass and types of muscle fibres

Muscles represent about 35–40% of body weight in a healthy adult and are composed mainly of type I, IIA, and IIX fibres (Bottinelli et al. 1999; Bottinelli and Reggiani 2000; Lee et al. 2000). The amount of muscle mass, as well as its metabolism, are determinants of energy expenditure in adults. In fact, muscle mass can explain 20–30% of total oxygen consumption at rest and therefore of body metabolism. Interestingly, differences in muscle metabolism can explain up to 50% of the variability in energy expenditure between individuals. Unlike other tissues with high energy expenditure, such as the kidney or the brain, total muscle energy expenditure is modifiable because both muscle mass and composition are modifiable (Zurlo et al. 1990). Promoting greater energy expenditure at baseline and over 24 h of the day prevents weight gain and the accumulation of adipose tissue, reducing the risk of obesity (Ravussin et al. 1988; Zurlo et al. 1990).

Muscle is the main carbohydrate store in the body, both in response to insulin and to contractile activity (DeFronzo 1988; Lund et al. 1995). In postprandial conditions, the muscle can capture up to 70% of ingested glucose (DeFronzo 1988), mainly in fibres type I and IIA, which are more oxidative, rich in mitochondria, with higher expression of the facilitated glucose transporter member 4 (GLUT-4) and greater sensitivity to insulin than fibres type IIX (Daugaard et al. 2000; Levin et al. 2007; Mackrell and Cartee 2012). A higher percentage of type IIX fibres is found in obese subjects with IR than in thin subjects. In adults, insulin sensitivity and the elasticity of arteries are positively associated with the proportion of type I fibres, but this proportion is negatively associated with blood pressure. Together, these results suggest a relationship between fibre types, obesity, IR, and vascular function (Tanner et al. 2002; de Courten et al. 2015; Stegen et al. 2015; Fisher et al. 2017).

The ability of the muscle to accumulate carbohydrates is so important that alterations in glucose uptake in the thigh cause IR in humans (Olsen et al. 2005). The fact that voluminous and mainly oxidative muscles (i.e., up to 75–85% of type I and IIA fibres) are present in this region makes it quantitatively important for glucose homeostasis (Staron et al. 2000; Olsen et al. 2005; Londono et al. 2012).

Type I and IIA fibres also have a greater capacity to store lipids than type IIX fibres (Daugaard et al. 2000; Levin et al. 2007; Mackrell and Cartee 2012). This ability of the muscle to store lipids, intra- and extramyocellularly (Goodpaster et al. 2001; Li et al. 2015), gives it a role in the distribution of body fat (Sinha et al. 2002). This muscle storage of fat is relevant because it can lead to the development of regional IR (Jacob et al. 1999; Virkamaki et al. 2001; Savage et al. 2019; Kim and Kim 2021) and alter muscle oxidative metabolism (Petersen et al. 2004; Li et al. 2016).

In summary, the reservoir capacity of muscle, which depends on the amount of muscle and its composition, regulates whole-body glycemic and lipid metabolism, substrate oxidation, and body energy expenditure. Thus, increased muscle mass, with a phenotype with more oxidative fibres (I and IIA) is metabolically and hemodynamically healthier.

### The muscle is an endocrine organ which modulates cardiometabolic health

Myokines are mainly peptides and proteins produced and secreted by the muscle under different stimuli, with autocrine, paracrine, and endocrine functions that regulate the metabolism of various tissues (Ahima and Park 2015; Narvaez-Sanchez et al. 2019; Bay and Pedersen 2020). The myokine profile produced depends, among other factors, on muscle mass and composition. Although it has not been demonstrated, muscle quality is probably also a determinant of the circulating myokine profile of a human.

Several myokines have notable metabolic functions (e.g., modulation of glucose and lipid metabolism) and cardiovascular functions (e.g., regulation of vascular reactivity, production of nitric oxide, blood pressure, and angiogenesis), which give them an important role in the pathophysiology and development of some chronic cardiometabolic diseases.

*Irisin:* It reduces hyperglycemia, IR, and weight in mice fed a high-fat diet (Bostrom et al. 2012). Its metabolic functions are mediated in part by the induction of mitochondrial uncoupling in adipose tissue and muscle (Bostrom et al. 2012; Vaughan et al. 2014). In addition, it increases the expression of GLUT-4 and the mitochondrial content in myoblasts (Vaughan et al. 2014), favoring a more oxidative and healthier phenotype in this tissue.

Irisin is reduced in obese patients, in whom it was also positively associated with dysfunction of endothelium-dependent vasodilation (Hou et al. 2015). Irisin increased nitric oxide synthase phosphorylation and nitric oxide production in human and rat endothelial cells, and in aortic rings from obese mice, in a dose- and time-dependent manner (Han et al. 2015; Hou et al. 2016). The favorable vascular impact of irisin could also be because it improves the function of perivascular adipose tissue (Hou et al. 2016, 2017). Irisin has anti-inflammatory properties in many experimental models (Han et al. 2015; Hou et al. 2017; Mazur-Bialy et al. 2017), which can explain the metabolic improvement and vascular function induced by this myokine.

Although it is still debated, studies in humans tend to show an increase in serum irisin after continuous exercise at not less than 60% of the maximal physical capacity, interval training with peaks over 90% of the maximal physical capacity, or strength training over 60% of one maximal repetition, at least 3 times per week for more than 8 weeks (Dinas et al. 2017). However, the use of inadequately validated measurement techniques has limited the scope of the findings.

Therefore, the evidence suggests that irisin has beneficial metabolic and vascular effects in healthy humans and patients with chronic cardiometabolic conditions.

*Myonectin:* It is produced mostly, but not exclusively, by type I fibres. In murine models, it reduces circulating triglycerides and free fatty acids by increasing the expression of its transporters in the liver and fat tissue (Seldin et al. 2012). In addition, it increases in response to aerobic exercise in murines (Seldin et al. 2012). In agreement with these results, knockout mice for myonectin accumulated less triglycerides in the liver and showed an increase in lipid storage in hypertrophied adipocytes, as well as intolerance to an oral lipid load (Little et al. 2019).

The relevance of these results to humans is unclear. On the one hand, studies have shown a direct association between myonectin and IR markers, but contrary to expectations, no correlation was observed between myonectin and plasma lipids or total fat mass (Toloza et al. 2018; Mi et al. 2019). On the other hand, a negative association between serum myonectin and IR markers and visceral obesity was reported in another population (Li et al. 2021). Future studies should aim to better understand the role of myonectin in the pathophysiology of various diseases in humans.

*Apelin:* This myokine, also an adipokine, circulates in several isoforms. However, due to technical limitations, it is not always easy to know which isoform is responsible for the observed effects. In general, apelin is reduced with aging and with the loss of muscle mass in murines and humans (Vinel et al. 2018). Its supplementation in murine models increases muscle strength and mass, demonstrating an autocrine effect (Vinel et al. 2018). Therefore, it seems to have an important regulatory role in muscle status. Its expression increased in human muscle after an 8-week intervention consisting of cycling or running 5 times a week at 85% of the maximal physical capacity, and this increase was associated with an improvement in insulin sensitivity (Besse-Patin et al. 2014). Its effect is mediated by the activation of 5′-AMP-activated protein kinase (AMPK) and serine/threonine protein kinase akt (AKT) in muscle and adipose tissue (Dray et al. 2008; Yue et al. 2010). Given that apelin induces phosphorylation of nitric oxide synthase in several tissues, a vasodilator effect and therefore a beneficial cardiovascular effect have been observed (Tatemoto et al. 2001), confirming that this protein is beneficial for cardiometabolic and muscular health.

*Interleukins*: The skeletal muscle produces a wide panel of interleukins (Ostrowski et al. 1999; Steensberg et al. 2000, 2002; Quinn et al. 2002, 2011; Pedersen et al. 2003; Nielsen et al. 2007; Bay and Pedersen 2020). In general, this cocktail has beneficial effects on health, as it favors glucose metabolism, is lipolytic, anti-inflammatory, immunomodulatory, and favors muscle hypertrophy, opposing the induction of IR and indirectly improving vascular function.

*Chitinase-3-like protein 1 (CHI3-L1)*: It is produced and secreted by immune, endothelial and muscle cells. It is expressed at lower levels in the muscle tissue of overweight subjects with glycemic alterations (Gorgens et al. 2016; Kwak et al. 2020), and increases in muscle and serum after a single bout of continuous exercise at 70% of the maximal physical capacity or strength training at 80% of one maximal repetition (Gorgens et al. 2016; Kwak et al. 2020). Its anti-inflammatory effects and its ability to induce the activation of AMPK and AKT as well as the translocation of GLUT-4 in muscle cell lines explain its beneficial effect on insulin sensitivity and myoblast proliferation (Gorgens et al. 2016; Kwak et al. 2020). In humans with coronary disease and in murine models, CHI3-L1 reduces and stabilizes the atherosclerotic plaque (Tsantilas et al. 2021), granting this myokine a clear cardiovascular and metabolic protective profile.

*Musclin:* It reduces glucose uptake and glycogen synthesis, both in the presence and in the absence of insulin, in animal models, and cultured myotubes (Nishizawa et al. 2004), inducing IR. In this sense, musclin reduces the phosphorylation of AKT induced by insulin (Liu et al. 2008). Elevated insulin activates AKT, which phosphorylates forkhead box protein O1 (FoxO1), producing a loss of suppression of the musclin gene, thus linking excess insulin, typical of an IR state, to an excess of musclin, favoring a vicious cycle that generates more IR (Nishizawa et al. 2004; Sierra et al. 2016). It is upregulated by palmitate, an inductor of IR, in cellular models (Gu et al. 2015; Guo et al. 2019) and is mostly expressed in type II fibres (Banzet et al. 2007), which predominate in obese and diabetic patients (Tanner et al. 2002). Musclin is increased in murine models fed a high-fat diet (Deng and Tang 2012; Yu et al. 2016; Chen et al. 2017) but is reduced in serum and muscle as a response to an exercise program based on ladder climbing or swimming during 8 to 13 weeks (Deng and Tang 2012; Shimomura et al. 2021).

In humans, we have shown a positive association between serum musclin and IR in adults with metabolic syndrome (Sanchez et al. 2021). In addition, musclin positively correlated with insulinemia and visceral fat (Sanchez et al. 2021) and was reduced after interventions with either continuous exercise on a treadmill at 60% of the maximal physical capacity or interval training with peaks over 90% of the maximal physical capacity for 12 weeks (Gallo-Villegas et al. 2022), validating the findings described above in murine models. In summary, musclin seems to be a key player in the induction of metabolic alterations.

Figure 1 summarizes the mechanisms by which a better muscle status protects from cardiometabolic diseases and mortality.

## Methods to quantify the CRF and muscle status

### Cardiorespiratory fitness

It can be assessed by measuring VO_2max_ in maximum stress tests (direct maximum tests with gas analysis) or estimated with maximum stress tests (indirect maximum tests without gas analysis), submaximal field and clinical tests, and nonexercise prediction equations (Cooper 1968; Bruce et al. 1973; Jackson et al. 1990; Laboratories 2002; Maranhao Neto Gde et al. 2004; Balady et al. 2010; Nes et al. 2011; Jackson et al. 2012; Kaminsky et al. 2019b; Cuenca-Garcia et al. 2022).

The direct tests combine procedures of the conventional stress test with ventilatory analysis of expired gases, which allow the concomitant evaluation of three functional variables with prognostic value: (i) VO_2_; (ii) carbon dioxide production (VCO_2_); and (iii) minute ventilation (VE) (Balady et al. 2010). VO_2max_ corrected for body mass is the gold-standard measure of physical cardiorespiratory capacity, while the slope of the VE/VCO_2_ ratio is a key indicator of ventilatory efficiency, which is abnormally high in most patients with cardiovascular or pulmonary diseases (Balady et al. 2010; Arena et al. 2014). Although the performance of the direct maximal stress test involves the participation of trained human resources, as well as expensive equipment, given the independent and additive information for the diagnosis and prognosis of many patients, its use may be justified (Balady et al. 2010; Arena et al. 2014). In many clinical environments, it is increasingly feasible to perform the direct stress test to quantify VO_2max_ as a measure of CRF. There are reference values of VO_2max_ calculated from population data of different countries (Peterman et al. 2020). Recently, a process was initiated to develop global reference standards for VO_2max_ obtained from direct maximum stress tests (Peterman et al. 2020; Kaminsky et al. 2022).

Through indirect tests, CRF can be estimated from regression equations according to the speed, inclination, duration, and workload (in watts) reached on a treadmill or cycle ergometer (Bruce et al. 1973). When the CRF is estimated using a treadmill protocol, the tests should be performed without allowing the patients to lean on the handrails. Likewise, care must be taken in the appropriate selection of the protocol according to the functional capacity of each individual.

Submaximal tests on a treadmill, bicycle, or step can estimate the CRF from the response of the heart rate to the intensity of the exercise (Davies 1968). Additionally, there are field exercise tests or submaximal clinical tests to estimate the CRF with regression equations that take into account the distance covered in 12 min (Cooper test) and six minutes (six-minute walk test) or the time to travel 1.5 miles (Cooper 1968; Laboratories 2002). These tests are highly reliable in adults (intraclass correlation coefficient, ICC > 0.9) (Cuenca-Garcia et al. 2022) and provide valuable information for clinical practice and should be considered when evaluating large groups of resources are limited. However, they are not as accurate as the direct stress tests to estimate CRF.

Nonexercise prediction equations are an alternative to maximal and submaximal stress tests to estimate CRF in the health setting (Jackson et al. 1990, 2012; Maranhao Neto Gde et al. 2004; Nes et al. 2011; Kaminsky et al. 2019b; Arcila et al. 2022). These models include physiological variables commonly evaluated that indicate physical fitness, such as: (i) sex, (ii) age, (iii) body mass index, (iv) heart rate at rest, and (v) physical activity performed (objective and subjective) (Jackson et al. 1990; Maranhao Neto Gde et al. 2004; Arcila et al. 2022). The nonexercise prediction equations to estimate CRF have shown consistent associations with mortality from all causes and of cardiovascular origin and have good discriminatory power and excellent capacity for risk reclassification (Stamatakis et al. 2013; Qiu et al. 2021). They could be a practical, quick, economical, safe, first-line tool for evaluating CRF and predicting cardiovascular risk in primary health settings, which is why their use is suggested for follow-up in adults at least once a year (Ross et al. 2016; Kaminsky et al. 2019b). Different nonexercise prediction equations have been validated in different regions of the world (Jackson et al. 1990; Maranhao Neto Gde et al. 2004; Nes et al. 2011; Jackson et al. 2012; Ross et al. 2016; Kaminsky et al. 2019b; Arcila et al. 2022).

Care takers should consider the use of submaximal, clinical, or field tests as an alternative because these can tell them how the individual responds to exercise and what exercise to prescribe (Kaminsky et al. 2019b). Finally, patients with chronic diseases should undergo a direct maximal stress test (Ross et al. 2016). Table 1 shows the median and interquartile range for the distribution of VO_2max_ values obtained from direct and indirect maximum tests on the treadmill for selected ages (Kaminsky et al. 2015).

**Table 1:** Reference values of VO _2max_ (mL O _2_.kg ^−1^.min ^−1^) obtained in treadmill directly and indirectly according to the FRIEND study

Age (years)	Direct		Indirect	
	Male	Woman	Male	Woman
	Median (interquartile range)^a^ mL O_2_.kg^−1^.min^−1^		Median (interquartile range)^a^ mL O_2_.kg^−1^.min^−1^	
30–39	42.4 (35.9–49.2)	30.2 (25.3–36.1)	42.4 (37.8–47.0)	36.7 (32.0–41.0)
40–49	37.8 (31.9–45.0)	26.7 (22.1–32.4)	40.1 (35.9–44.9)	34.5 (30.2–38.6)
50–59	32.6 (27.1–39.7)	23.4 (19.9–27.6)	37.1 (32.8–41.8)	31.4 (28.0–35.2)
60–69	28.2 (23.7–34.5)	20.0 (17.2–23.8)	33.8 (29.5–38.3)	28.8 (25.1–32.3)

^a^The first value corresponds to the 25th and the second to the 75th percentile. Adapted from: Kaminsky et al. 2015

### Muscle status

The most common strength and functional tests in adults include handgrip strength, standing and sitting in a chair, isometric mid-thigh pull and gait speed. They are reliable (ICC > 0.85), inexpensive, fast and easy to apply (Bohannon et al. 2010; Cooper et al. 2014; Leong et al. 2016; Cruz-Jentoft et al. 2019; Ramirez-Velez et al. 2021; Cuenca-Garcia et al. 2022). Typically, men have grip values up to 50% higher than those of women, and there are notable differences between races. In general, grip strength increases up to age 25–30, stabilizes between 30 and 40 years, and then shows a curvilinear reduction (Schlussel et al. 2008; Dodds et al. 2014; Leong et al. 2016; Lee et al. 2019). The high variability has necessitated the adoption of different reference values for both sexes in different regions of the world over a wide age range (Leong et al. 2016; Martinez-Torres et al. 2022). The PURE study reported reference values for the general population of at least 21 countries of different levels of development. Table 2 shows the medians and the 25th and 75th percentiles of the data obtained in various regions of the world (Leong et al. 2016). In addition, multiple countries have seen recent specific studies that report normative values with selected percentiles of grip strength for adults between the fourth and ninth decades of life (Schlussel et al. 2008; Dodds et al. 2014; Wong 2016; Steiber 2016; Wang et al. 2018; Landi et al. 2020; Ramirez-Velez et al. 2021). Those patients below the 25th percentile are at greater risk. The ease of measurement, the availability of reference values, and their relevance to clinical outcomes make feasible the evaluation and routine follow-up of grip strength in primary healthcare settings. For instance, during the medical consultation of patients with cardiometabolic risk factors, or when registering participants in exercise, physical activity or conditioning interventions, nutritional programs or physiotherapy sessions.

**Table 2:** Reference values of grip strength (kg) for different regions in the world according to the PURE study

Age (years)	South America		Europe/North America		Middle East		China	
	Male	Woman	Male	Woman	Male	Woman	Male	Woman
	Median (interquartile range)^a^ kg		Median (interquartile range) kg		Median (interquartile range) kg		Median (interquartile range) kg	
30–39	45 (39–52)	29 (23–33)	50 (43–56)	30 (26–35)	45 (40–51)	26 (22–30)	45 (40–50)	28 (24–32)
40–49	43 (37–50)	27 (21–31)	49 (42–56)	30 (25–34)	43 (38–48)	25 (22–29)	43 (37–48)	28 (23–32)
50–59	41 (33–46)	25 (21–29)	46 (39–52)	27 (23–31)	40 (35–46)	23 (20–27)	40 (34–45)	26 (22–29)
60–69	37 (31–44)	23 (19–27)	42 (36–47)	25 (21–29)	35 (31–40)	21 (18–24)	36 (31–41)	23 (20–27)

^a^The first value corresponds to the 25th percentile and the second to the 75th percentile. Adapted from: Leong et al. 2016

The chair test consists of measuring the time required for the patient to stand and sit five times, without using the support of their hands or arms. The results depend on age and sex and are correlated with the strength of the lower limbs. The average times increase from 6 to 14 s between the third and ninth decades of life (Bohannon et al. 2010; Cruz-Jentoft et al. 2019; Landi et al. 2020; Gao et al. 2021). Values in percentiles for some populations are also available (Landi et al. 2020; Gao et al. 2021). Recent results suggest that both bilateral and unilateral isometric mid-thigh pull tests can be used to measure lower-limb strength with high reliability in healthy and obese subjects (Orange et al. 2019; Grgic et al. 2022). Isometric mid-thigh pull strength partially explains the performance of these populations in functional tasks (Orange et al. 2019; Grgic et al. 2022) and is expected to be increasingly used among middle-age and older adults in the near future. Gait speed is typically evaluated over 4 m. Walking velocities greater than 1.0 m/s are expected (Cruz-Jentoft et al. 2019).

Though nuclear magnetic resonance (NMR) and computed tomography are the reference methods, dual-energy X-ray absorptiometry (DXA), bioelectrical impedance analysis (BIA), and anthropometry are the most used methods to study muscle mass.

DXA is the first-choice method because provides accurate (low variability between measurements: ICC 0.97 and coefficient of variation, CV < 1.0%) and valid (comparable with the reference test) total and appendicular lean mass information in a noninvasive, fast, safe, and economical way (Rubbieri et al. 2014; Cruz-Jentoft et al. 2019; Walowski et al. 2020; Kawakami et al. 2021; Aristizabal et al. 2021). Fat-free adipose tissue (FFAT) corrections of the DXA lean mass measurements are currently necessary. They can be performed assuming that ~ 13–15% of the adipose tissue is a fat-free component (Abe et al. 2019, 2020a). The application of this correction increases validity when assessing muscle mass, without adding costs (Abe et al. 2019, 2020b), thus strengthening DXA as a tool for evaluating muscle status. The appendicular mass index seems to be the best indicator and the most used to assess muscle mass (Cruz-Jentoft et al. 2019; Walowski et al. 2020). Unfortunately, the normative values still lack the FFAT correction, and there are doubts about the feasibility of comparing data obtained in multiple countries: there are variations of up to 35% in the cutoff values between different populations using the same technique (Walowski et al. 2020).

Although simple, cheap and useful in large surveillance studies, classical BIA and anthropometry have limited validity for their application in muscle mass evaluation in individual cases (Rubbieri et al. 2014; Furushima et al. 2017). Their reduced reliability and the lack of normative values further limit their use for following up on a specific patient in most health settings (Walowski et al. 2020). In addition, complex anthropometric models (Londono et al. 2012) are out of the expertise of most health care practitioners and are discouraged from general use.

Fortunately, improved methods such as specific bioelectrical impedance vector analysis (BIVA), refined anthropometric equations or equations integrating both BIA results and anthropometric measurements have partially overcome these limitations. If DXA is not available even after referral or if contraindicated, these techniques can be used as second-line choices to assess muscle mass.

Specific BIVA is based on a corrected classical bioelectrical impedance vectorial approach. It is cheap and effective to distinguish adults with different muscle mass index values. Further, reference data for some populations is available (Buffa et al. 2014). Anthropometric equations with enhanced validity (R^2^ over 0.90 and standard errors of the estimate as low as 1–2 kg compared to DXA or NMR) have been developed for total and appendicular mass measurements in some populations, including middle-aged adults (Al-Gindan et al. 2014; Lee et al. 2017; Heymsfield et al. 2020; Kawakami et al. 2021). These equations rely on simple measurements such as height, body weight, and a few circumferences and skinfolds. Others have refined equations to estimate total and appendicular muscle mass starting from BIA measurements with high validity (ICC over 0.96 and errors within 2 kg compared to DXA) in adults and elder subjects (Lee et al. 2018). Enhanced-validity equations should now be developed for different populations so its use can be spread soon.

Muscle composition can be studied in tissue obtained by biopsy, but since this is an invasive method, its use is not recommended. An alternative is the use of proton magnetic resonance spectroscopy (^1^H NMRS), which has been used in healthy subjects and in patients with metabolic syndrome and diabetes (Stegen et al. 2015; Gallo-Villegas et al. 2022; Vega et al. 2022). However, its use is not yet widespread because it is a costly, highly specialized technique. Similarly, the assessment of muscle quality based on quantifying myosteatosis by computed tomography or ^1^H NMRS (Larsen et al. 2020; Kim and Kim 2021) is currently limited to research studies.

## Application of concepts to clinical practice

*History of the present illness*: A 47-year-old Latino man who works in financial activities in an office is seen in an outpatient consultation. He comes for a biannual consultation as part of the cardiometabolic risk program, which he has participated in for 3 years. He reported deterioration in his functional capacity since the last consultation, which manifested as dyspnea and tachycardia when climbing stairs without angina or dizziness.

*Past medical history*: Overweight for 20 years. Four years ago, he started medication for fasting hyperglycemia (metformin 250 mg every 12 h) and arterial hypertension (losartan 50 mg every 24 h).

*Habits:* He is sedentary 6–8 h a day, has not performed physical activity in his free time for approximately 15 years, does not smoke, does not consume liquor, sleeps 7 h a day, and reports a high consumption of processed foods.

*Physical examination* reveals a good general condition. Vital signs sitting: heart rate 87 beats/min; respiratory rate 17 cycles/min; blood pressure 148/94 mmHg. Weight 89 kg; height 1.77 m; body mass index 28.4 kg/m^2^; waist circumference 98 cm. Normal heart sounds, conserved vesicular murmurs without superimposed sounds, and no carotid murmurs. Normal abdomen, normal peripheral pulses. Normal gait. Reduction of the force in the flexion–extension of the knee and hip. No myalgia.

*Physical tests in the office:* Grip strength of 33.5 kg by dynamometry in his dominant hand; 10.5 s to stand and sit in a chair five times; walking speed 1.2 m/s; estimated VO_2max_: 28.5 mL O_2_/kg/min (8.1 METs).

*Paraclinical:* Fasting glycemia 108 mg/dL, homeostatic model assessment (HOMA-IR) 2.8, glycosylated hemoglobin 6.1%, total cholesterol 210 mg/dL, HDL-cholesterol 40 mg/dL, LDL-cholesterol 172 mg/dL, triglycerides 175 mg/dL, creatinine 1.1 mg/dL, normal hemoleukogram. FFAT-corrected appendicular lean mass 18.9 kg, FFAT-corrected appendicular lean mass index (appendicular skeletal mass/height^2^) 6.1 kg/m^2^, visceral fat 820 g, and body fat percentage 42%, by DXA.

*Diagnoses*: Overweight and abdominal obesity (code XS7R, ICD-11), arterial hypertension grade 1 (BA00), fasting hyperglycemia (5A40), metabolic syndrome and insulin resistance (5A44), dyslipidemia (5C8Z), decreased muscle mass (FB329), low cardiorespiratory fitness (CM91), and sedentary lifestyle (QE20).

*Decision:* An endurance training program plan with cardiovascular components and muscle strengthening was prescribed under current guidelines. Referral to the nutritionist and psychologist. Pharmacological treatment was adjusted to achieve goals.

*Follow-up:* Six months later, the patient improved his VO_2max_ by 12%, increased his grip strength by 7%, lost 2.6% of body fat, and gained 660 g of appendicular muscle mass and 1.0 kg of total lean mass. His fasting glycemia was 100 mg/dL, HbA1c 5.8%, HOMA-IR 2.5, and blood lipids were 10% better. He was recommended to continue with the multidisciplinary treatment.

**Fig. 2:**
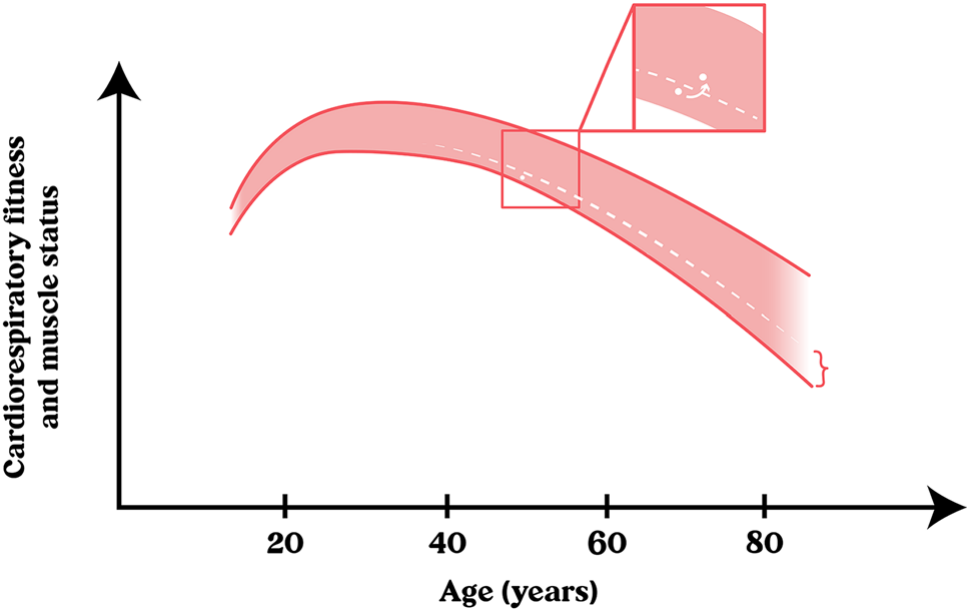
General evolution of cardiorespiratory fitness and muscle status during lifetime. Both conditions rapidly increase during youth, then stabilize during early adulthood, and finally decrease with slow, more or less linear kinetics. There is always variability in the values found across all ages, from which those in the lowest quartile (below percentile 25th, P25, i.e., below the white, discontinuous line) are at increased risk of cardiometabolic morbidity and mortality. The white dot illustrates the position of the patient in the clinical case regarding the distribution of the population of the same age. The routine evaluation of cardiorespiratory fitness and muscle status in adults allows to identify those below the P25 and take effective actions to help them soon cross the line over the P25 (white arrow in the insert), notably reducing their cardiometabolic risk

*Discussion of the clinical case*: The patient has metabolic, cardiorespiratory, and muscular alterations (Fig. 2). Previous management was limited to a pharmacological approach, without considering lifestyle changes, such as nutritional and physical activity modifications. Basic evaluations were lacking at the time of patient admission to the cardiometabolic risk program, such as body composition, VO_2max_, grip strength, and gait speed. An adequate follow-up and an early intervention would have improved the health of the patient before.

His CRF is poor since his VO_2max_ is within the lowest quartile according to that expected for his age and sex (10 METs; 35 mL O_2_/kg/min) (Kaminsky et al. 2015). The low CRF observed may be related to the lack of physical activity in his free time and his sedentary lifestyle. Taking into account that VO_2max_ was estimated by a nonexercise prediction equation, it is recommended to perform a supervised maximal or submaximal stress test to estimate VO_2max_ and evaluate the cardiorespiratory response to physical effort and thereby make a more precise exercise prescription.

The patient showed a grip strength in the lowest quartile according to the PURE study and also according to recent normative tables for the Latino population (Leong et al. 2016; Ramirez-Velez et al. 2021). His muscle mass is lower than the mean values expected for Latino men (Aleman-Mateo and Ruiz Valenzuela 2014; Aristizabal et al. 2021). This puts him at greater risk of developing cardiometabolic diseases compared to the general population, although he does not meet the criteria for the formal diagnosis of sarcopenia (Cruz-Jentoft et al. 2019).

This analysis highlights that appropriate cutoff values should be applied to each individual, that the objective of the evaluation of strength and muscle mass goes beyond seeking the diagnosis of sarcopenia, and that this middle-aged patient can reduce his cardiometabolic risk through interventions aimed at improving his CRF and muscle status.

The main actions that increase the CRF and protect the muscle are a healthy diet, well-prescribed exercise, and prevention of chronic or inflammatory conditions (Argiles et al. 2016; Cruz-Jentoft et al. 2019). The first item was addressed by referring the man to a nutritionist, who may suggest him a low-energy diet. Low-energy (i.e., caloric restriction and intermittent fasting) or very low-carbohydrate diets improve cardiometabolic health and extend healthspan and lifespan in humans and model organisms (Stekovic et al. 2019; Cipryan et al. 2022). These diets are characterized by their provision of no more than 1200 kcal/day or 50 g/day of carbohydrates, respectively, to induce rapid weight (1.0–2.5 kg/week) and fat loss. To preserve muscle mass and function under these conditions, it is recommended to keep: (i) slower rates of weight loss; (ii) higher protein intakes; (iii) incorporate strength training as part of the exercise program. Indeed, the prescribed exercise for this patient included an endurance intensity component and a strength component, following international recommendations (Bull et al. 2020). Considering that a low-energy diet and exercise have an anti-inflammatory effect, both interventions can be used in the prevention and treatment of chronic and inflammatory conditions (Pedersen 2017; Stekovic et al. 2019; Cipryan et al. 2022). Pharmacological treatments were adjusted to achieve the clinical objectives. Finally, a psychologist addressed the personal and occupational psychosocial factors of the patient linked to his sedentary lifestyle and unhealthy chronic behaviors (Seefeldt et al. 2002; Sassen et al. 2010; Godoy-Izquierdo et al. 2021). Patient’s exposure to health-damaging behaviors and, potentially, chronic stressful experiences, may have led him to an increased allostatic load. A quantitative assessment of allostatic load can be done based on biomarkers and clinical (physical and psychosocial) signs and symptoms. This will allow to understand if the patient is coping well enough with his life situations or may have reached an allostatic overload condition, further increasing his cardiometabolic risk (Fava et al. 2019; Guidi et al. 2021). The aim is to offer the patient a multidisciplinary, comprehensive, personalized evaluation and intervention, which assures long-lasting improvements.

These improvements are gradual and take 3 to 6 months, as observed in the follow-up. The beneficial change in body composition (less fat mass, more lean mass) and in the biochemical profile, and the gains in VO_2max_, strength, and muscle mass guarantee a reduction in his risk of morbidity and mortality. The observed increase in METs, as part of the CRF of the patient, is in agreement with what was expected (Batacan et al. 2017; Su et al. 2019) and lowers his risk of death from cardiovascular causes by 15% (Kodama et al. 2009). The greatest benefits are reached by people with greater physical cardiorespiratory deconditioning (< 5 METs) when they begin to be physically active (Eijsvogels et al. 2016). Similarly, the patient exceeded the goal of a ≥ 1% increase in global and appendicular lean mass, enough to improve his lipid and glycemic control and reduce his risk of developing metabolic syndrome (Oh et al. 2021).

## Proposal for translation

**Fig. 3:**
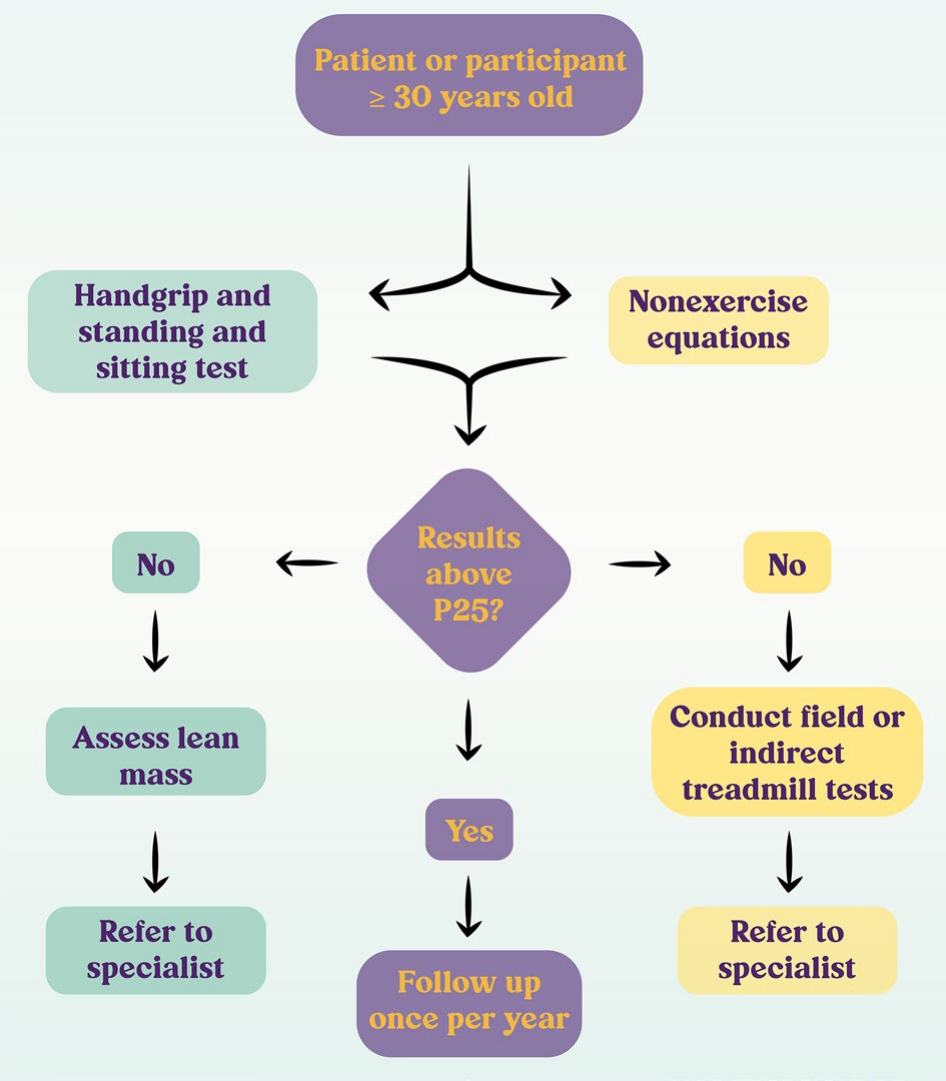
Proposed flowchart for the implementation of the routine evaluation of the cardiorespiratory fitness and muscle status in adults. A wealth of epidemiological, mechanistic and methodological evidence supports that patients or participants from the general population seeking assistance in different primary health scenarios such as clinics, nutrition centres, training and conditioning centres, and personalized health programmes, who are ≥ 30 years old, should be evaluated for their cardiorespiratory fitness (right) and muscle status (left). Start by estimating the VO_2max_ using nonexercise equations and assess muscle status through handgrip and standing and sitting tests. Compare results with normative values according to the subject`s population. If above percentile 25th (P25), reevaluate in one year. If the performance was below P25, complement the evaluation and refer to a medical specialist, who should take action to improve both cardiorespiratory fitness and muscle status so that they surpass P25

The feasibility of applying several techniques in non-specialized health contexts, along with the possibility offered by them to classify the patients above or below the percentile 25th (P25) based on the published normative data, allow us to propose a simple flowchart to guide the implementation of the routine evaluation of the CRF and muscle status in adults (Fig. 3). The external validity of the epidemiological evidence presented along this review makes this approach suitable for any patient or individual older than 30 years of age from the general population who contacts a primary health carer.

## Limitations and perspectives

Given the lack of longitudinal studies to generate normative values and given that the benefit is greater than the risk, it is sensible to use cross-sectional studies to evaluate and monitor physical capacity. Validation studies of the nonexercise prediction equations should be done to estimate VO_2max_ in different populations, with a view to using them in the first level of health care. Unfortunately, the emphasis of the literature on the diagnosis of sarcopenia has caused much information from population groups to be reported only with means and cutoff values of 2 or more standard deviations (Wigodski et al. 2019; Lee et al. 2019; Alrashdan et al. 2021; Gonzalez et al. 2021), losing the utility for assessment, monitoring, and early intervention in the general population. The way to measure grip strength should still be better standardized and unified around the world. Great efforts should be made to generate FFAT-corrected normative values of lean mass, particularly of the appendicular mass index, and they should be presented in percentiles. More studies on muscle quality should be carried out to generate simple measurement protocols and reference values for different populations worldwide. The future development and standardization of the biochemical measurement of some myokines will open the door to the evaluation of muscle endocrine function as part of the battery tests to assess the muscle status more comprehensively. It is necessary to estimate in longer longitudinal studies the impact of a gain in physical capacity on cardiometabolic outcomes and mortality. People living with disabilities (physical, mental, sensory, or intellectual) deserve special attention because they are at a greater risk of injury and of developing non-communicable chronic diseases and age-related health conditions at earlier ages. Those people have lower physical fitness and poorer health than the general population. In these adults, it is a priority to assess their CRF and muscle status, closely follow them up and implement intervention strategies to improve their condition (Martin Ginis et al. 2021). Health schools around the world are invited to include training modules on the importance of evaluating CRF and muscle status in the general population. This should be accompanied by a strategy to boost practitioners’ skills at assessing the results of paraclinical tests and adequately prescribing measures that improve the altered parameters.

In health and care settings, a recent study of the implementation of routine measurement of grip strength in the elderly showed that it is cheap and can be successful when there are highly motivated advisors and instructors who are integrated with health personnel with whom they have a shared commitment. High turnover of personnel involved in the implementation strategy should be avoided (Ibrahim et al. 2018). Despite this progress, it is clear that the loss of functional reserve and the deterioration in CRF and muscle status begin decades before a person reaches old age. The knowledge derived from these pioneering experiences will allow us to extend the implementation of the measures discussed here to other healthcare settings from early adulthood (Fig. 3), improving the morbidity and mortality of the general population.

## Conclusions

Many high-quality studies show that CRF and muscle status are determinants of morbidity and mortality in multiple population groups over a wide range of ages in both sexes, justifying the generalization and applicability of the results to those ≥ 30 years old. Because they are robust indicators of health status, with prognostic value, CRF and muscle status should be routinely evaluated in primary health practice. The main parameter that reflects CRF is VO_2max_. The best and more practical indicators of muscle status are grip strength, appendicular mass, and performance in functional tests such as sit-and-stand test. The availability of protocols and techniques that are easy to apply allows us to start evaluating CRF and muscle status at the first level of health care without further delay. Epidemiological evidence permits us to propose P25 as a cutoff to take actions. The evaluation of the muscle status must not be limited to the assessment of the diagnostic criteria for sarcopenia.

## Data Availability

Not applicable.

## Abbreviations

AKT: Serine/threonine protein kinase akt
AMPK: 5′-AMP-activated protein kinase
avO_2_: Arteriovenous oxygen difference
BIA: Bioelectrical impedance analysis
CHI3-L1: Chitinase-3-like protein 1
CV: Coefficient of variation
DXA: Dual-energy X-ray absorptiometry
FoxO1: Forkhead box protein O1
GLUT-4: Solute carrier family 2, facilitated glucose transporter member 4
ICC: Intraclass correlation coefficient
IR: Insulin resistance
MET: Metabolic equivalents
NMR: Nuclear magnetic resonance
Q: Cardiac output
VCO_2_: Carbon dioxide production
VE: Minute ventilation
VO_2max_: Maximal oxygen uptake
^1^H NMRS: Nuclear magnetic resonance spectroscopy
